# Genome-Scale Phylogenetic and Population Genetic Studies Provide Insight Into Introgression and Adaptive Evolution of *Takifugu* Species in East Asia

**DOI:** 10.3389/fgene.2021.625600

**Published:** 2021-02-22

**Authors:** Bo Liu, Zhixiong Zhou, Yulin Bai, Junyi Yang, Yue Shi, Fei Pu, Peng Xu

**Affiliations:** ^1^Fisheries Research Institute of Fujian, Xiamen, China; ^2^Fujian Key Laboratory of Genetics and Breeding of Marine Organisms, College of Ocean and Earth Sciences, Xiamen University, Xiamen, China; ^3^Xiamen Key Laboratory of Urban Sea Ecological Conservation and Restoration, Xiamen, China

**Keywords:** *Takifugu* species, phylogenetic, introgression, hybridization, adaptive evolution

## Abstract

As a typical marine adaptive radiation species, most *Takifugu* species are widely distributed in East Asian offshore, which have diversified morphological characteristics and different ecological habits. The phylogenetic relationship and population structure of the *Takifugu* species was complicated because of incomplete lineage sorting, widespread hybridization and introgression. Therefore, to systematically clarify the phylogenetic relationships of *Takifugu* genus, explore the introgression and natural hybridization between different *Takifugu* species, and detect the selective signatures in the adaptive evolution of diversified traits, whole-genome resequencing was used in 122 *Takifugu* samples from 10 species. Phylogenetic analysis showed solid sister-group relationships between *Takifugu bimaculatus* and *Takifugu flavidus*, *Takifugu oblongus*, and *Takifugu niphobles*, *Takifugu rubripes*, and *Takifugu obscurus*, *Takifugu xanthoptreus*, and *Takifugu ocellatus*. Further admixture analysis indicated the divergence of *T. obscurus* population and the bidirectional gene flow between *T. bimaculatus* and *T. flavidus*. Using species-specific homozygous genetic variance sites, we detected the asymmetric introgression between *T. bimaculatus* and *T. flavidus* at China East sea and southern Taiwan Strait. By genome-scale genetic diversity scanning, we detected two copies of *syt1*, *zar1* and *tgfbr1* related to the semilunar reproduction rhythm in *T. niphobles*, involved in memory formation, embryo maturation and female reproduction. Furthermore, we also found lots of *T. niphobles* specific mutations in CDS region of circadian rhythm related genes and endocrine hormone genes. For *Takifugu* species, our research provides reliable genetic resources and results for the phylogeny, introgression, hybridization and adaptive evolution, and could be used as a guide for the formulation of the protection and proliferation release policies.

## Introduction

Adaptive radiation leadsP to the evolution of different species with diverse ecological features and phenotypes within a rapidly multiplying lineage. Hybridization and gene introgression are the two main driving forces of adaptive radiation (Schluter, 2000) since they increase genetic diversity and enhance the ability of a species to quickly occupy a new ecological niche (Marsden et al., 2011; Todesco et al., 2016; Quilodrán et al., 2020). Conversely, hybridization is also considered as “genetic pollution,” which will accelerate the extinction of native species (Simon et al., 2020). Moreover, human activities are impacting the genetic makeup of an increasing number of native species (McFarlane et al., 2020). On the other hand, frequent gene exchange makes elucidating the lineage relationships between species complicated, and thus, it is difficult to identify their lineages and evolutionary history accurately through a few genes or the mitochondrial genome. Therefore, the phylogenetic relationships and evolutionary history of species that have arisen from adaptive radiation need to be explored on a genome-wide scale. Cichlid fish in East Africa have undergone one of the largest radiations and given rise to approximately 2,000 known species (Seehausen, 2006). The widespread incomplete lineage sorting (ILS), hybridization, gene introgression, and the complicated lineage relationships among cichlids were studied and elucidated on a genome-wide scale (Brawand et al., 2014; Meier et al., 2017; Malinsky et al., 2018). In addition, species that have arisen from adaptive radiation have high genetic similarity, exhibit diverse phenotypes, and are excellent specimens for the study of evolutionary developmental biology and evolutionary genomics (Macfadden and Hulbert, 1988). Using comparative genomics and population genetics, many studies have identified candidate genes underlying phenotypic differentiation among species are a product of adaptive radiation, such as dog (Akey et al., 2010), cichlid fishes (Torres-Dowdall et al., 2017) and finch (Tebbich et al., 2010).

*Takifugu* species are marine species that have arisen from adaptive radiation and are widely distributed in East Asian offshore. They belong to the family *Tetraodontidae* and the order Tetraodontiformes. Fatal toxicity and swelling body in danger are typical features of these species. In contrast to cichlid fish, only about 25 *Takifugu* species have undergone explosive speciation during Pliocene (Santini et al., 2013). However, they have diverse morphological characteristics, in terms of body size and coloration, and different ecological habits, such as spawning behavior and adaptability to freshwater (Kato et al., 2010; Motohashi et al., 2010). For example, *Takifugu rubripes* have a larger body size and faster growth rate than those of others (Hosoya et al., 2013). During the rising tides in the evening and around the days of the full moon, *Takifugu niphobles* spawn in intertidal beaches (Yamahira, 1994). Moreover, *Takifugu obscurus* and *Takifugu ocellatus* will migrate to the Yangtze River during the breeding season to spawn, and several differences in reproductive strategies between these two pufferfishes have been reported (Yang and Chen, 2008). Hence, *Takifugu* species are considered to be an excellent model for evolutionary studies of adaptive radiation in marine and estuarine environments.

Similar to cichlid fish, several natural and artificial hybridization studies of *Takifugu* species have been conducted and have reported the absence of absolute reproductive isolation (Takahashi et al., 2017). In addition, the hybridization of *T. rubripes*, *T. obscurus*, and *T. flavidus*, under artificial conditions, were reported to result in fertile offspring, which indicated that there were no reproductive isolation between these species (Jiang et al., 2016; Yoo et al., 2018). These types of hybridizations and introgression can contribute to phenotypic diversification, however, they also complicate the phylogenetic relationship between different *Takifugu* species. On the other hand, rapid speciation results in ILS, which is another obstacle in accurately describing phylogenetic relationships. Therefore, it is necessary to comprehensively explore phylogenetic relationships between the *Takifugu* species on a genome-wide scale. Compared with other vertebrate species, *Takifugu* species have the smallest genome of approximately 400 Mb, which is half the size of the cichlid genome and less than *Gasterosteus aculeatus* (Zhou Z. et al., 2019). This compact genome facilitates genetic research in *Takifugu* species. As the first sequenced teleost genome, the genome of *T. rubripes* was published in 2002 (Aparicio et al., 2002). To date, three other chromosome-level genomes have been published, including that of *T. bimaculatus*, *T. flavidus*, and *T. obscurus* (Zhou Y. et al., 2019; Zhou Z. et al., 2019; Kang et al., 2020). However, only a few studies involving phylogenetic and interspecific hybridization analysis in *Takifugu* species exist and are based on the mitochondrial genome and amplified fragment length polymorphisms (AFLP) (Yamanoue et al., 2009; Takahashi et al., 2017). In addition, there are only a few population genetic studies involving multiple *Takifugu* species to date (Zhang et al., 2020). The publication of high-quality genomes motivates population genetics and comparative genomic studies, which are crucial to the understanding of phylogenetic relationships, evolutionary histories, and adaptation strategies of the different *Takifugu* species during explosive speciation (Nielsen et al., 2009).

The primary aim of this study was to establish a phylogeographical relationship between 10 *Takifugu* species along the Chinese coast, including Yellow Sea, Eastern China sea, and Taiwan strait using the whole genome re-sequencing method for genotyping. Using this database, we identified reliable phylogenetic relationships and potential events of hybridization and introgression among these populations. In addition, we scanned the genome and detected several different genes in *T. niphobles* that are associated to the semilunar reproduction rhythm in the intertidal zone, and they are involved in memory formation, embryo development, and rhythm regulation, respectively. To the best of our knowledge, this is the first comprehensive phylogenetic and population genomics study of *Takifugu* species on a genome-wide scale. The results provide important clues for understanding the genetic divergence, hybridization, introgression, and speciation of marine species that have arisen from adaptive radiation in East Asia.

## Materials and Methods

### Sampling and DNA Extraction

**FIGURE 1:**
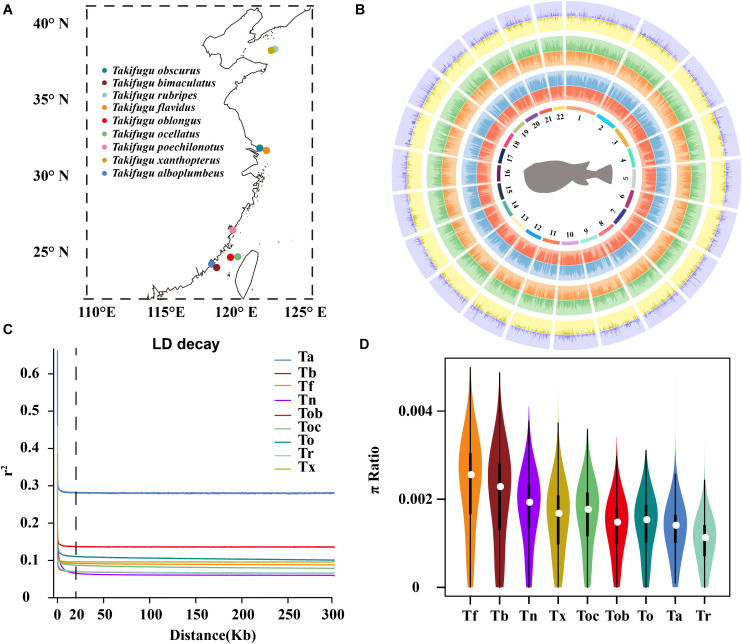
**(A)** The geographic locations of sample collection. **(B)** Circos histogram of the statistic of genetic variation calling. The tracks from inside to outside are 22 chromosomes, SNP number (red), indel number (blue), intergenic SNP number (orange), intergenic INDEL number (green), number of SNPs in CDS (yellow), and number of INDELs in CDS (purple). **(C)** The LD decay of 10 *Takifugu* species. **(D)** Genome-wide distribution of nucleotide diversity in a 50 kb non-overlapping window. The white dot was median, black rectangle represented the interquartile range, the black line represented the 95% confidence interval.

Individuals from nine *Takifugu* species, consisting of *T. obscurus* (To), *T. bimaculatus* (Tb), *T. rubripes* (Tr), *T. flavidus* (Tf), *T. oblongus* (Tob), *T. ocellatus* (Toc), *T. poechilonotus* (Tp), *T. alboplumbeus* (Ta), and *T. xanthoptreus* (Tx), were sampled from marginal seas, straits, and estuaries along the Chinese coastal line during the summer and autumn in 2018, with two sites in the north Yellow sea (Tr, Tx), two sites in the estuary of the Yangzi river (To, Tf), one site in the China East sea (Tp), and four sites in the Taiwan Strait (Tob, Toc, Ta, Tb) (Figure 1A). In total, 70 fish were collected, and their tail fins were stored in 95% ethanol before genomic DNA extraction. All samples were collected following national legislation.

Genomic DNA was extracted from the tail fin as described previously (Zhou Z.X. et al., 2019). Briefly, approximately 10 mm^2^ of the fin samples were shredded and lysed at 56°C with 600 μL of a solution, consisting of STE buffer (10 mM of Tris-HCl, 10 mM of NaCl, 25 mM of EDTA), 10% SDS buffer, and 0.2 mg proteinase K. Subsequently, equal volumes of Tris-phenol and chloroform/isopropanol (24:1) were successively added to extract the DNA. The mixture was incubated for 1 h at −20°C for precipitation and the DNA was collected after centrifugation for 15 min. After washing twice with 70% ethanol, genomic DNA was resuspended in Milli-Q water, and RNase was added. The purified genomic DNA was run on a 1% agarose gel, using gel electrophoresis, and stained with GelGreen to confirm its genomic integrity. Next, DNA concentrations were quantified using a Qubit fluorometer 4.0 (Thermo Fisher Scientific, Waltham, MA, United States), and then diluted to 10 ng/μL for genotyping. Finally, all 70 samples were subjected to Whole-Genome Re-Sequencing.

### Genome Resequencing and Genotyping

The sequencing libraries were constructed using the TruePrep DNA Library Prep Kit V2 for Illumina (Vazyme Biotec, Nanjing, China). Whole-genome re-sequencing was performed on the Illumina Hiseq X Ten platform at the Novogene Corporation, Beijing, China. Paired-end reads from each individual were aligned to the reference genome Tb (SWLE00000000) using the MEM algorithm of Burrows-Wheeler Aligner (BWA) (Li and Durbin, 2009). Samtools v1.8 and PICARD v2.18.9 were used to convert the binaries and sort files (Li et al., 2009). GATK v4.0.5.2 was employed to genotype all individuals, using standard procedures and preliminary filters for genetic variation sites with the parameters of “QD < 5.0 || FS > 35.0 || MQ < 55.0 || SOR > 3.0 || MQRankSum < −12.5 || ReadPosRankSum < −8.0 > “(McKenna et al., 2010). Finally, VCFTOOLS v0.1.06 was used to strictly filter low-quality sites with the parameters of “-mac 2 -min-alleles 2 -max-missing-count 2” (Danecek et al., 2011). To identify the sites of genetic variance in the mitochondrial genome, the same protocol and parameters were executed with the reference mitochondrial genome of *T. bimaculatus* (KP973944.1). Due to the insufficient sample size of Tp, two individuals were removed from evaluations of linkage disequilibrium (LD) and nucleotide diversity. LD decay was investigated for the nine species by PopLDdecay (Zhang et al., 2019).

### Phylogenetics, Genetic Structure Analysis, and Identification of Hybridization and Introgression Events

Jmodeltest (2-2.1.10) was used to identify the best model for phylogenetic tree construction (Posada, 2008). Two maximum-likelihood trees were constructed using RAxML v8.2.12 with GTR + I + G model and 1000 bootstrap replicates, based on 236,794 single nucleotide polymorphisms (SNPs) on fourfold degenerate synonymous sites (4DTv) and 15,666 SNPs in the mitochondrial genome, respectively (Stamatakis, 2014). Principal component analysis (PCA) and structure analysis with all the SNPs were performed using GCTA v1.26.0 and ADMIXTURE v1.3.0, respectively (Alexander et al., 2009; Yang et al., 2011). The 3D plots and structure column charts were drawn using scatterplot3d in R.

After filtering the potential introgressive hybridized *T. bimaculatus* and *T. flavidus* individuals, we identified the *Takifugu* species-specific genetic variation sites among 10 species, which fixation index (*F*_st_) were 1 between one Takifugu species and other individuals. ADMIXTURE was used to estimate the introgressive level of eight potential hybrid individuals from the Tb and Tf populations, and 851 species-specific genetic variation markers were identified between the remaining individuals from the Tb and Tf populations. To further estimate the levels of introgression of the remaining 28 Tb and Tf individuals, we used 31,131 highly differentiated markers (*F*_st_ > 0.8) between the Tb and Tf individuals. We scanned the whole genome for complete differentiation sites between Tob and Tx-Toc individuals, rather than using the sliding window scanning method for *F*_st_. This is because weak gene exchange signals in a short region may be masked by numerous noise sites. The allele frequency was calculated using PLINK (v1.90b6.16) and all Manhattan scatterplots were drawn using R (Purcell et al., 2007).

### Detecting Genome Regions and Candidate Genes Selective to the Semilunar Reproduction Rhythm

To estimate selective genes to the semilunar reproduction rhythm in Takifugu *niphobles* (Tn), we first scanned the genome using *F*_st_ and Nucleotide diversity (*π*) Ratio with a sliding window size of 50 Kb and step of 10 Kb. The average ratio of the *π* ratio of other *Takifugu* species and that of Tn [average(πx/πTn)] was used to represent the difference in nucleotide diversity. We identified the regions with the highest 1% of *F*_st_ values (*F*_st_ > 0.225) or a significant difference in nucleotide diversity (| Log_2_average(πx/πTn)| > 1.5). All candidate genes were annotated by BLAST in the NCBI database^1^. Fine map scanning was performed using *F*_st_, *π* ratio, and Tajima’s *D* with a sliding window size of 1 kb and a step of 500 bp.

## Results

### Genetic Diversity Between *Takifugu* Species

We re-sequenced the genome of 70 wild *Takifugu* individuals and downloaded the re-sequencing data of an additional 52 individuals from NCBI, who belonged to 10 different *Takifugu* species, to estimate sites of genetic variation, phylogenetic relationships, potential hybridization and introgression, and the presence of selective signatures of adaptive evolution in the different species. A total of 663.7 GB of sequence data was collected, which is equivalent to 5.4 GB of data for each individual, with an average depth of approximately 13.5 (Supplementary Tables 2, 3). The data were mapped to their respective reference and mitochondrial genomes, and the average mapping ratio, genome coverage, and sequencing depth was 98.40%, 93.43%, and 13.21 X, respectively (Supplementary Table 4). Tb and Tf genomes showed the highest mapping ratio of 98.47%, 95.73%, and highest average genome coverage of 98.53% and 95.60%, for Tb and Tf, respectively (Supplementary Table 5). Furthermore, except for Toc12, the genome coverage of all other samples was greater than 90% (Supplementary Table 4). The interspecific universality of the reference genome in *Takifugu* species indicated similarity of genomes and a close phylogenetic relationship between them. After quality control, 13,598,656 SNPs and 1,629,677 insertion and deletion mutations (INDELs) were identified across 22 chromosomes (Figure 1B and Supplementary Table 6). The average length of INDELs was 5.7 bp and ranged from 1 bp to 259 bp, and the average distance between adjacent SNPs and INDELs was 27 bp and 225.7 bp, respectively (Supplementary Table 6). After careful classification of all sites of genetic variation, 55.21% SNPs and 53.52% INDELs were found to be in the intergenic region, and 7.74% SNPs and 1.18% INDELs were found to be in the coding sequence (CDS; Figure 1B and Supplementary Table 6). In the mitochondrial genome, 1,566 SNPs and 12 INDELs were identified, of which 8.23% SNPs and 75% INDELs were in the intergenic region (Supplementary Table 6).

Multiple linkage maps of the *Takifugu* genome showed a higher recombination rate compared with other vertebrates (Kai et al., 2005, 2011; Shi et al., 2020), and this was supported by our findings. In addition to Ta, all *Takifugu* species showed a very low degree (*R*^2^ < 0.2) of LD when the window of analysis was greater than 20 kb, indicating active recombination in the *Takifugu* genome (Figure 1C). Nucleotide diversity reflects the degree of polymorphism within a population. Using a 50 kb sliding window on a genome-wide scale, we found that Tf and Tb had the highest number of polymorphisms, which may be related to frequent introgressions between them (Figure 1D). In addition, Tr showed a reduced nucleotide diversity compared to that in other species. This reduction is likely the result of successive artificial inbreeding over generations and a decline in the wild population size (Nakajima et al., 2008).

**FIGURE 2:**
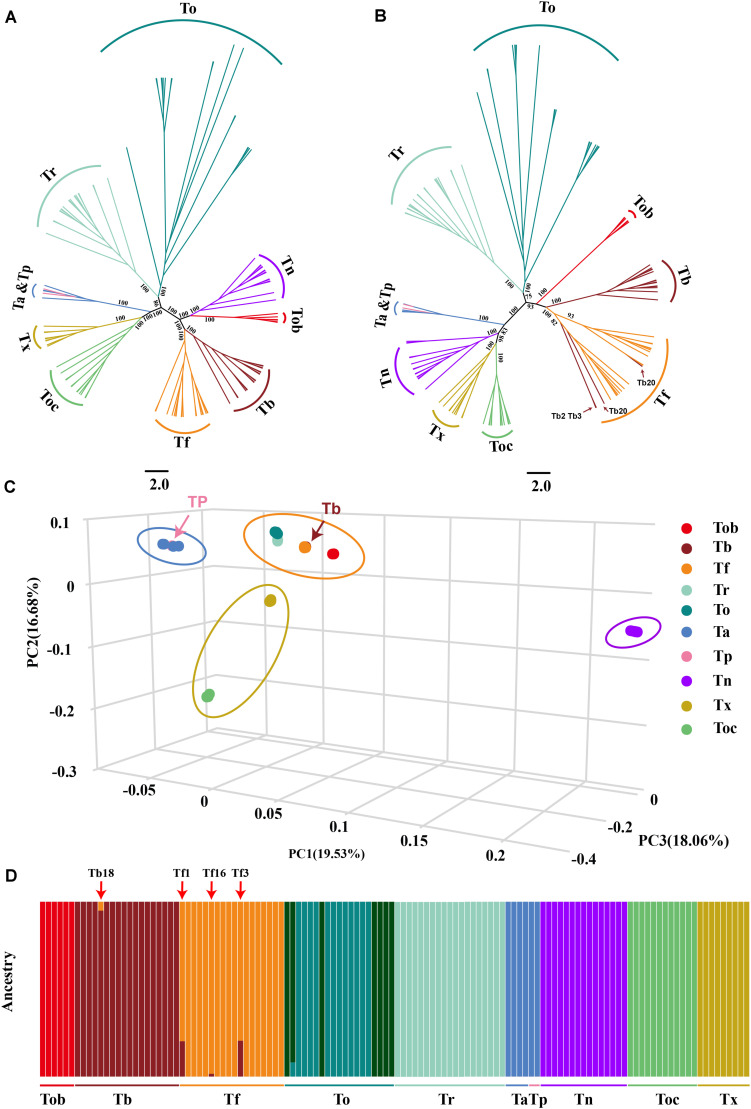
Population structure and relationships of 10 *Takifugu* species. **(A)** Maximum-likelihood tree of the relationships between the 10 *Takifugu* species (122 individuals) based on 4DTv sites. The bootstrap value of the main branches was listed on the branches. **(B)** Maximum-likelihood tree of the relationships between the 10 *Takifugu* species (122 individuals) based on mitochondrial genetic variation sites. The bootstrap value of the main branches was listed on the branches. **(C)** 3D plot visualizing the principal component (PC) analysis. **(D)** The proportion of ancestry for each individual assuming 10 ancestral population (*K* = 10). Colors in each columnar represent the likelihood proportion of a *Takifugu* genome assigned to a source population and each population is labeled at the bottom.

We identified the complete interspecific differentiation sites of genetic variation and thus removed potential hybrid individuals (Tb2, Tb3, Tb20, Tb22) (Figures 2A,B). Finally, we identified 980,788 species-specific SNPs and 135,292 species-specific INDELs. Tn exhibited the highest number of species-specific genetic variation sites, with 271,722 SNPs and 35,273 INDELs, and Tf and Tb exhibited the least number of species-specific genetic variation sites, with only 165 SNPs and 18 INDELs in Tf and 174 SNPs and 10 INDELs in Tb, indicating a possible close relationship during evolutionary history. Similarly, Ta and Tp exhibited a few numbers of species-specific genetic variation sites, with only 285 SNPs and 54 INDELs in Ta and 479 SNPs and 95 INDELs in Ta, which is due to the close relationship and small sample size between them. For other species, 96,246 SNPs and 12,832 INDELs were species-specific in To, 233,998 SNPs and 32,325 INDELs were species-specific in Toc, 93,955 SNPs and 14,214 INDELs were species-specific in Tr, 194,716 SNPs and 27,253 INDELs were species-specific in Tob, 89,048 SNPs and 13,218 INDELs were species-specific in Tx (Table 1).

**TABLE 1:** Statistics of species-specific genetic variation sites.

Species	Specific-SNP	Specific-INDEL
To	96246	12832
Toc	233998	32325
Tr	93955	14214
Tob	194716	27253
Tf	165	18
Ta	285	54
Tb	174	10
Tp	479	95
Tn	271722	35273
Tx	89048	13218
Total	980788	135292

### Phylogenetics and Structure Analysis

Considering that *Takifugu* is a product of adaptive radiation and is thus affected by natural selection, the accuracy of phylogenetic trees is reduced due to convergent evolution and ILS of the species (Jarvis et al., 2014; Chen et al., 2019). Hence, we used fourfold degenerate synonymous sites (4DTv) in the autosomes and SNPs in the mitochondrial genome to construct phylogenetic trees. In the 4DTv phylogenetic tree, sister-group relationships were confirmed between Tb-Tf, Tob-Tn, Tr-To, and Tx-Toc. In addition, four Ta individuals and two Tp individuals belonged to the same group, which indicated that these two species were closely linked (Figure 2A). In the wild, Tp and Ta individuals have very similar morphological patterns and almost overlapping habitats in the Northwest Pacific region (Supplementary Figure 1). Given our insufficient sample size, we cannot conclude whether these two species are synonymous to each other. Most topologies of the mitochondrial phylogenetic tree were consistent with that of the 4DTV phylogenetic tree. Interestingly, the Tn group was closer to the Tx-Toc sister-group, but Tn and Tob formed a reliable sister-group in the 4DTv phylogenetic tree. In previous studies, the close relationship between Tn and Tx-Toc sister-group was demonstrated in phylogenetic trees of the pufferfish based on the sequence of *cytb*, *12sRNA* (Zhang and He, 2008) and the whole mitochondrial genome (Zhang and He, 2008; Yamanoue et al., 2009). In addition, four *T. bimaculatus* individuals were clustered in the Tf group (Figure 2B). The different phylogenetic relationships between mitochondria and autosomes indicated that hybridization played an important role in the evolutionary history of *Takifugu* species and hybrid progenies could survive in nature. PCA analysis further confirmed the phylogenetic relationship revealed by the two phylogenetic trees. Based on the three main principal components (percent cumulative eigenvalue variance = 54.27%; Figure 2C), the 10 *Takifugu* species were divided into four major groups, including the To-Tr-Tf-Tb-Tob group, the Ta-Tp group, the Tx-Toc group, and the Tn group.

To further understand the genetic structure in these *Takifugu* populations, we used the ADMIXTURE tool to analyze genetic variation sites with *K* values ranging from 3 to 18, wherein *K* is the assumed number of ancestral populations. The coefficient of variation suggested that *K* = 10 is the most likely number of genetically distinct groups within our samples (Supplementary Figure 2). Ta and TP showed the indistinguishable genetic group. To populations had two genetic groups that were not detected in other species, which indicated that there may be two populations with large genetic differences in To. In addition, we found four other potential hybrid individuals (Tb18, Tf1, Tf3, and Tf16), which indicated that a bidirectional gene flow existed between Tb and Tf (Figure 2D).

### Interspecific Introgression and Hybridization

Two closely related species, Tb and Tf, coexisted across a vast area, from the southern East China Sea to the southern Yellow Sea (Supplementary Figure 3). Previous studies have shown that hybrid individuals of Tb and Tf existed in the East China Sea (Yin et al., 2016). However, there is no record of hybridization between Tb and Tf individuals in the southern Taiwan Strait. Through phylogenetic analysis of 4DTv and mitochondrial genome, we not only found the hybridization between female Tf and male Tb individuals was excited in nature but also extended the zone wherein hybridization occurs to the southern Taiwan Strait. In the phylogenetic analysis, we detected four potential hybrid individuals (Tb2, Tb3, Tb20, and Tb22). Except for them, we identified 143 SNPs in the mitochondrial genome and 15 SNPs in autosomes that *F*_st_ = 1 between Tb and Tf (Supplementary Figure 4A). In the mitochondrial genome, only Tb22 had one Tb-specific SNP, and all the remaining SNPs of four hybrid individuals were absolutely consistent with the genotype of Tf (Supplementary Figure 4B). In autosomes, Tb2 and Tb20 had a homozygous Tf-specific SNP (Supplementary Figure 4A). It suggested that both parents of Tb2 and Tb20 were Tf-specific homozygous or heterozygous on the respective loci.

**FIGURE 3:**
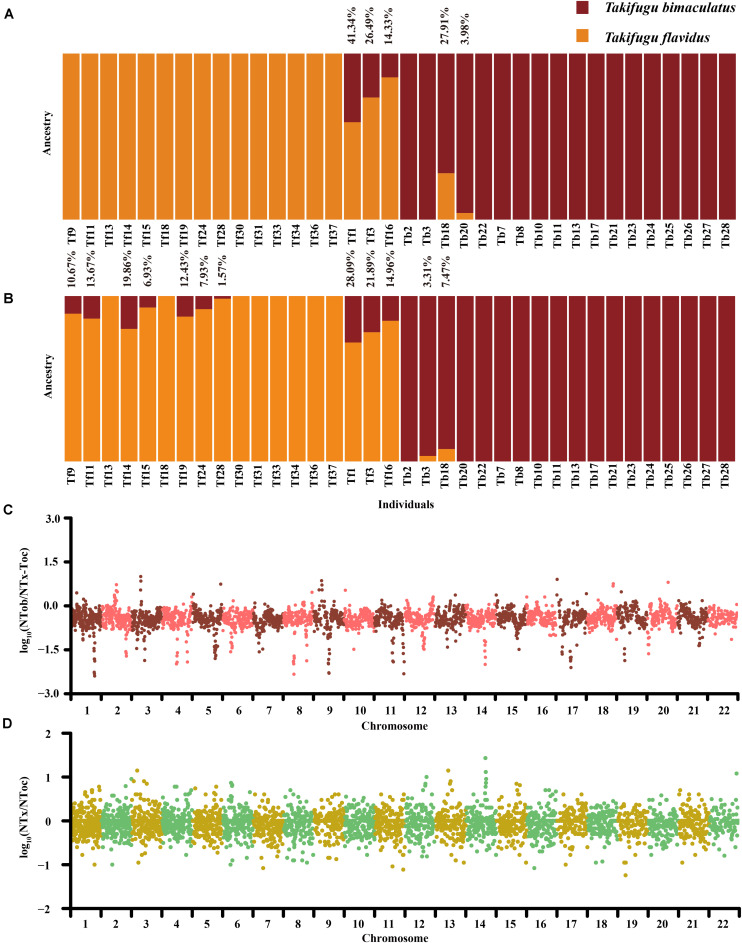
**(A)** Admixture analysis based on 851 homozygous genetic variation sites on autosomes between Tb and Tf. **(B)** Admixture analysis based on 31,131 homozygous genetic variation sites on autosomes between Tb and Tf. The orange represented the genetic group of Tf. The claret represented the genetic group of Tb. The introgressive ratio was listed on column. **(C)** The Genome scanning of genetic similarity between Tob and Tx-Toc in Tn. The ordinate is the Log_10_ of the ratio of Tob-specific homozygotes site number to Tx-Toc homozygotes site number. **(D)** The Genome scanning of genetic similarity between Tx and Toc in Tn. The ordinate is the Log_10_ of the ratio of Tx-specific homozygotes sites number to Toc homozygotes site number.

To further evaluate the bidirectional gene flow in the Tb-Tf sister group, we re-detected sites of species-specific genetic variation between Tb and Tf in 28 “purebred” individuals, which were pedigrees in the phylogenetic and admixture analysis (excluding Tb2, Tb3, Tb18, Tb20, Tb22, Tf3 and Tf16). A total of 851 sites of genetic variation in the autosomes and 129 SNPs in the mitochondrial genome were identified and these were all homozygous and completely differentiated (*F*_st_ = 1) in the Tb-Tf sister group. Based on the 129 mitochondrial SNPs, four Tb individuals (Tb2, Tb3, Tb20, and Tb22) showed consistency genetic group with the Tf population, suggesting that these individuals had a hybrid background of female Tf and male Tb (Supplementary Figure 5). In contrast, all Tf individuals had pure mitochondrial genetic components. Based on the 851 SNPs and INDELs in the autosomes, three Tf individuals showed varying levels of introgression (14.33–41.34%) from Tb. Only two Tb individuals showed varying levels of introgressive (3.98% and 27.91%) from Tf (Figure 3A). In addition, we used 31,131 SNPs and INDELS on autosomes and 135 SNPs in the mitochondrial genome, which were highly differentiated in 28 “purebred” individuals of Tb and Tf (*F*_st_ > 0.8), to explore whether there was a weak introgression in these “purebred” individuals that showed no introgression signal by the phylogenetics and structure analysis in 122 Takifugu individuals. Results showed that there was low-level introgression in seven “purebred” Tf individuals with introgression rate ranging from 6.93 to 19.86%, but no introgressive signal was detected in the “purebred” Tb individuals (Figure 3B). Moreover, Tf14 showed a higher level of introgression than that of Tf16, and this was detected using ADMIXTURE on a genome-wide scale. For individuals with lower levels of introgression, a weak introgressive signal covered a large number of non-introgressive sites. With an increase in the frequency of backcrossing with the previous generation, the probability of detecting introgression declines. Hence, we did not rule out the possibility of genetic pollution in the remaining “purebred” individuals (Supplementary Figure 6). More importantly, as we relaxed the filtering standard of differentiated genetic variance sites between Tb and Tf, the impact of ILS on introgressive evaluation and the overshadowing effect of non-introgressive sites continued to expand. However, the significant difference between “purebred” Tb and Tf individuals indicated that there was widespread introgression from Tb to Tf at the junction of the Yellow Sea and the East China Sea. On the other hand, a reverse introgression existed at the southern Taiwan Strait.

In the phylogenetic analysis, the phylogenetic difference between mitochondrial genomes and autosomes of Tn indicated that the ancestors of Tn may be influenced by asymmetric introgression from Tob and Tx-Toc (Figures 2A,B). We investigated the genotypes and allele frequencies of 563,658 SNPs and 60,752 INDELs in the autosomes and 254 SNPs in the mitochondrial genome in all *Takifugu* species; all of which were homozygous and completely differentiated (*F*_st_ = 1) between Tob and Tx-Toc group. In contrast to the largest number of Tx-Toc-specific mitochondrial SNPs, Tn had the largest number of homozygous sites of Tob-specific genetic variance in the autosomes, consisting of 132,295 SNPs and 12,008 INDELs, and the major allele in 53% of heterozygous loci was Tob-specific (Supplementary Figure 7 and Supplementary Table 7). A 100 kb sliding window was used to identify the conserved regions with low recombination frequencies after introgression by estimating the ratio of homozygous SNPs between Tob and Tx-Toc (Figure 3C). In the Tn population, several conserved regions were further confirmed as sites of potential introgression. For example, on chromosome 9, two regions between 4.36 and 4.59 Mb were conserved in Tob individuals, but the highly conserved regions in Tx-Toc individuals were between 7.23 and 7.38 Mb (Supplementary Figure 8). These numerous differences of genetic origin were more likely to be caused by frequent autosomal recombination after potential introgression, rather than selective sweep or convergent evolution. To further explore the genetic origin of Tn loci exchanged from the Tx-Toc group, we used 578,210 markers of genetic variance that exhibited complete differentiation (*F*_st_ = 1) between Tx and Toc. Further, these loci were homozygous in the Tob genome to reduce any statistical bias caused by genetic drift. Finally, 28,705 specific homozygous loci were identified in the Tx and the Tn genomes, and 33,469 specific homozygous loci were identified in the Toc and the Tn genomes. In addition, in the Tn population, almost all specific homozygous loci identified in Tx and Toc individuals were evenly scattered throughout the genome, and several regions showed higher homology between the Tn and Tx genomes (Figure 3D). For example, Tn and Tx individuals shared almost all specific homozygous loci from 10.2 Mb to 10.7 Mb on chromosome 14, which also showed higher homology with Tx-Toc-specific loci than Tob-specific loci (Supplementary Figure 9). The enrichment of Tx-specific homozygous loci in this region showed that ILS and gene draft may also play important roles in the speciation process of *Takifugu*.

### Adaptive Evolution of the Semilunar Reproduction Rhythm in *T. niphobles*

**FIGURE 4:**
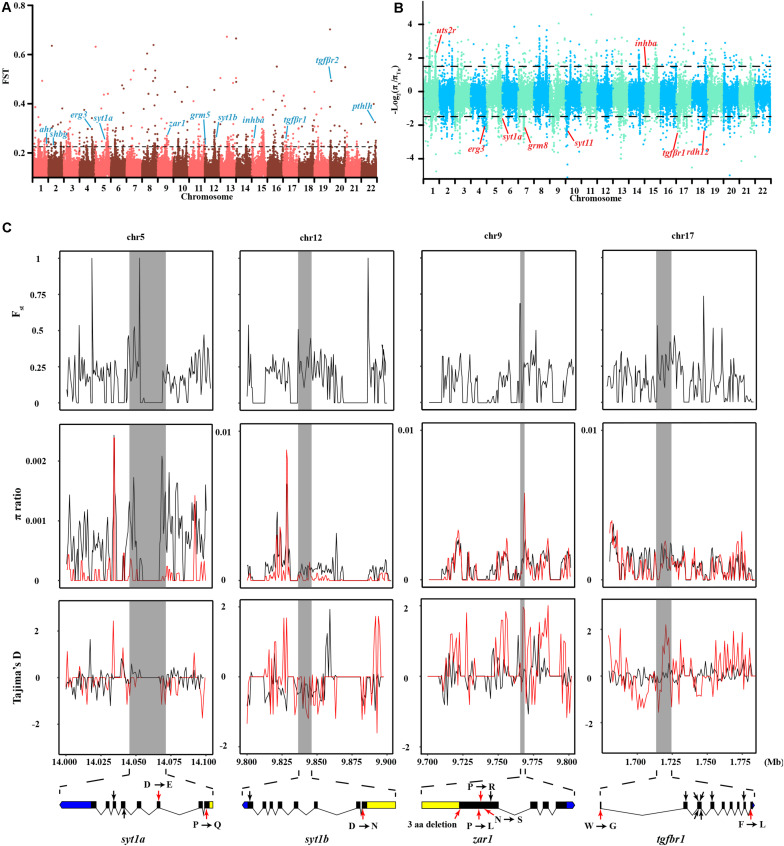
Genomic regions and candidate genes of *T. niphobles* under selected. **(A)** Selective scanning of *F*_st_ among Tn and other *Takifugu* species. *ahr*, aryl hydrocarbon receptor; *shbg*, sex hormone-binding globulin; *erg3*, early growth response protein 3; *sty1a*, synaptotagmin-1a; *zar1*, zygote arrest protein 1; *grm5*, metabotropic glutamate receptor 5; *sty1b*, synaptotagmin-1b; *inhba*, inhibin beta A chain; *tgfβr1*, TGF-beta receptor type-1; *tgfβr2*, TGF-beta receptor type-2; *pthlh*, parathyroid hormone-related protein. **(B)** Selective scanning of π ratio between Tn and other *Takifugu* species. *uts2r*, urotensin-2 receptor; *grm8*, metabotropic glutamate receptor 8; *sty11*, synaptotagmin-11; *rdh12*, retinol dehydrogenase 12. The candidate gene was marked in red on the Manhattan plot. **(C)** Selective scanning on four selection genes. The *F*_st_, π ratios and Tajima’s *D* values were plotted using 500 bp sliding windows. D, aspartic acid; E, glutamic acid; P, proline; Q, glutamine; R, arginine; L, leucine; N, asparagine; S, serine; W, tryptophane; G, glycine; F, phenylalanine. In π ratio plotting and Tajima’s *D* plotting, the red curved lines represented the Tn individuals and the black curved lines represented the residual individuals. Genome annotations are shown at the bottom [black bar: coding sequences (CDS); blue bar: 5′UTR; yellow bar: 3′UTR]. The boundaries of *syt1a*, *syt1b*, *zar1* and *tgfbr1* are marked in gray shadow. The red arrows represented nonsynonymous mutations, the black arrows represented synonymous mutations.

In contrast to other *Takifugu* species, Tn has specific reproductive habits, such as spawning in the intertidal zone during the rising tides, in the evening, around the days of the full moon (Yamahira, 1994). An experiment in an aquarium showed that Tn exhibited obvious aggregating behavior without tidal changes and also precisely maintained the semilunar reproductive rhythm during the spawning season (Motohashi et al., 2010). Hence, this reproductive rhythm is an innate social behavior in the Tn population. Moreover, different Tn populations prefer to spawn at particular beaches with an inclination angle of 8.84°, which is beneficial for gathering, stranding, and spawning (Yamahira, 1997). These social behaviors are closely related to the regulation of memory, rhythm, and levels of sex hormones. We compared the genetic differences between Tn and other *Takifugu* species to identify the genes specifically associated with spawning behavior. A total of 2,202 sliding windows showed selected signals, which were 17.16 Mb in size (Figures 4A,B and Supplementary Tables 8, 9). After functional annotation, we uncovered 15 candidate genes which are shown in Table 2. Among these, we found two copies of *syt1*, which encodes for Synaptotagmin-1, a calcium sensor that is responsible for calcium-triggered rapid release of neurotransmitters at the synapse (Geppert et al., 1994; Sudhof, 2013). *syt1* plays a key role in α-amino-3-hydroxy-5-methyl-4-isoxazole-propionic acid receptor (AMPA) mediated long-term potentiation (LTP) and participates in the process of learning and memory formation (Wu et al., 2017). Upon fine scanning of *F*_st_, π Ratio, and Tajima’s *D*, we detected sweep signals in the coding region of *syt1a* and *syt1b* (Figure 4C). In *sty1a*, 12 homozygous mutations were identified in the exons, consisting of two nonsynonymous mutations, two synonymous mutations, four mutations in the 3′UTR, and one mutation in the 5′UTR. The two nonsynonymous mutations changed proline to glutamine in exon 1 and aspartic acid to glutamic acid in exon 3. In *sty1b*, 16 homozygous mutations were identified in the exons, consisting of one nonsynonymous mutation, one synonymous mutation, three mutations in the 3′UTR, and eleven mutations in the 5′UTR. The nonsynonymous mutation on exon 1 changed aspartic acid to L-asparagine (Figure 4C and Supplementary Table 10). In addition, we also identified zygote arrest protein 1 (*zar1*), which is a critical maternal effect gene and participates in the process of oocyte-to-embryo transition (Wu et al., 2003). A study investigating embryo development in Tn reported shorter incubation periods and faster completion time in Tn than that of other *Takifugu* species (Sun et al., 2019). The shorter embryo maturation time of Tn may be due to a rapid change in tidelines around the days of full moon and new moon. In this study, we found significant differentiation signals around *zar1* on chromosome 9 in Tn compared to that of other *Takifugu* species. Nine homozygous mutations were identified in the exons, consisting of one nonsynonymous mutation, three synonymous SNP mutations, three amino acid deletions, two SNPs, one INDEL mutation in the 3′UTR, and one mutation in the 5′UTR (Figure 4C and Supplementary Table 10). Moreover, TGF-β receptor type-1 (*tgfbr1*), which is an essential gene for the female reproductive system, had a positive selection signal on chromosome 17. Nine homozygous mutations were identified in the exons, consisting of two nonsynonymous mutations and seven synonymous mutations. In addition to the above genes, we found that many rhythm-related genes and endocrine hormone genes in Tn had specific mutations in the CDS (Supplementary Table 11). Many of these genes have been reported to play a key role in the lunar-associated spawning of Tn. Transcription studies have shown that three types of gonadotropin-releasing hormone exhibited different expression levels in the hypothalamus during the reproductive cycle (Shahjahan et al., 2010). In addition, the nocturnal secretion of melatonin from the pineal gland may be involved in the transmission of photoperiodic signals from the moonlight to the hypothalamus in Tn (Ando et al., 2013). In conclusion, our results showed that genes involved in LTP, maternal effect, rhythm regulation, and endocrine hormone secretion had species-specific mutations in the Tn genome, providing new insights into the genetic evolution of intertidal reproductive behavior and semilunar spawning rhythm.

**TABLE 2:** Candidate gene selected of *T. niphobles*.

Chromosome	Start	End	*F*_st_	Log_2_(π_x_/π_Tn_)	Genes	*Abbreviation*
1	26530001	26540000	0.23	–0.09	Aryl hydrocarbon receptor	*ahr*
1	21835001	21845000	0.22	2.25	Urotensin-2 receptor	*uts2r*
2	8330001	8340000	0.23	–0.35	Sex hormone-binding globulin	*shbg*
4	15120001	15130000	0.3	–2.09	Early growth response protein 3	*egr3*
5	14035001	14045000	0.26	–1.56	Synaptotagmin-1a	*sty1a*
7	5845001	5855000	0.12	–2.14	Metabotropic glutamate receptor 8	*grm8*
9	9765001	9775000	0.27	–0.71	Zygote arrest protein 1	*zar1*
10	675001	685000	0.14	–2.57	Synaptotagmin-11	*sty11*
11	16205001	16215000	0.23	–1.20	Metabotropic glutamate receptor 5	*grm5*
12	9835001	9845000	0.29	–0.19	Synaptotagmin-1b	*sty1b*
15	845001	855000	0.25	1.53	Inhibin beta A chain	*inhba*
17	1715001	1725000	0.25	–2.37	TGF-beta receptor type-1	*tgfbr1*
18	13385001	13395000	0.21	–2.11	Retinol dehydrogenase 12	*rdh12*
20	13115001	13125000	0.55	–	TGF-beta receptor type-2	*tgfbr2*
22	8935001	8945000	0.32	–	Parathyroid hormone-related protein	*pthlh*

## Discussion

### Asymmetric Hybridization Between *T. bimaculatus* and *T. flavidus*

*Takifugu* species had arisen from adaptive radiation in the coastal area of East Asia during the Pliocene epoch (1.8–5.3 million years ago) (Yamanoue et al., 2009). Similar to other species that have arisen from adaptive radiation, reproductive isolation is not absolute among the different *Takifugu* species, and several studies involving natural and artificial hybridization experiments have demonstrated the fertility of the hybrid offspring (Takahashi et al., 2017). Although female mate choice and prezygotic isolation (behavioral or ecological) in the different *Takifugu* species may reduce the frequency of hybridization, the rate of introgression is accelerated by changes in the environment and the population size (Saetre et al., 2001).

In this study, we detected bidirectional introgression between the Tf and Tb genomes by admixture analysis. Although we did not detect an F1 hybrid individual in the natural environment, there is sufficient evidence to show that Tb and Tf coexist in the region spanning the Taiwan Strait to the southern Yellow Sea, and their hybrid progeny is fertile. In the Taiwan Strait, four hybrid progeny, from matings between male Tb and female Tf, were identified based on phylogeny, but very low levels of introgression were detected in these individuals, indicating that there were multiple backcrossings between female hybrid individuals and male Tb individuals after primary hybridization. In the junction of the Yellow Sea and the East China Sea, many Tf individuals exhibited an introgression signal from the Tb genome in their autosomes, but no introgression signal was detected in the mitochondrial genome, indicating that matings between male Tb and female Tf were the most common hybrid combination in this region. These asymmetric hybridizations are widespread across *Takifugu* species and are often based on the extremely rare existence of F1 hybrids (Wirtz, 1999; Takahashi et al., 2017). For the first time, we report two opposite asymmetric hybridizations in *Takifugu* species between different geographical regions, thereby providing an excellent model to explore the influence of environment and geographical conditions on natural hybridization.

Asymmetric hybridization between Tb and Tf can only be attributed to prezygotic causes, since the bidirectional introgression implied there was no reproductive isolation among Tb, Tf, and their hybrid progenies. The most direct explanation for asymmetric hybridization is the sexual selection hypothesis which states that the females of a rare species are more likely to choose the males of a dominant species than the same species mating, because mate choice becomes weaker in the absence of males of the same species (Takahashi and Takata, 2000; Cahill et al., 2015). In the south Taiwan Strait, Tb is the dominant species, and estuaries of the Zhang River and the Jiulong River, located near our sample points, are the important spawning grounds (Fang and Du, 2008). Tf is the rare species of this area, and there are no reports of Tf spawning in this area. Hence, the primary hybrid offspring often arose from matings between female Tf and male Tb individuals, and the F1 hybrids likely backcrossed with Tb. Therefore, due to the independence of maternal inheritance, the introgression signal in the autosome was rapidly weakened after multiple backcrosses, but the introgression in the mitochondrial genome was completely preserved in a few individuals.

On the other hand, the situation is more complicated in the junction of the Yellow Sea and the East China Sea. First, this is an important fishing area in China, and fisheries in this region have been severely declining (Zhu et al., 2017). Further, *Takifugu* is an economically valuable species and thus they have a small population size (Zhang et al., 2010). Under huge fishing pressure, the delicate balance between interspecific hybridization and conservative mate choice based on sufficient population size is disturbed. Consequently, many female Tf frequently reproduced with males from other *Takifugu* species, and this accelerated the speed of interspecific introgression in the autosomes (Mai et al., 2014). Another explanation is the possible northward expansion of the Tb population. Migration is one of the most important ways to cause introgression, and the distribution of species is greatly impacted by environmental changes (Nogue et al., 2009). For example, with the increase in global warming, grizzly bears have become more common on the Arctic island in Canada, and hybridizations between grizzly bears and polar bears have been reported in several regions (Pongracz et al., 2017). In marine fishes, the impact of global warming is more significant, changing the distribution, spawning season, and even the offspring sex ratios of damselfish (Donelson and Munday, 2015), cod (McQueen and Marshall, 2017) and *Argyrosomus coronus* (Potts et al., 2014). As the two biggest marginal seas, east of China, the sea surface temperature (SST) of the Yellow Sea and East China Sea displayed a sharp incline from 1980 to 2016 (Pei et al., 2017). This change may have facilitated the invasion of Tb in the northern East China Sea and the southern Yellow Sea. Last but not least, individuals that have escaped from artificial breeding enclosures may also be one of the catalysts for introgression between Tb and Tf. In the past two decades, with the success of artificial breeding of Tb and Tf, the aquaculture industry has rapidly developed around the coast of the China East Sea and Yellow Sea (Zhou Z. et al., 2019). In particular, Tf is widely cultured in the central and northern Taiwan Straits, and the northern border of artificial Tb breeding grounds is in the Bohai Sea, which is not its traditional habitat (Zhang et al., 2020). In addition, breeding programs of Tb and Tf also involve their hybridization (Jiang et al., 2016). Inevitably, some purebred individuals and artificial hybrids escape into the wild and invade the local population. In summary, the huge pressure exerted by the fisheries at the junction of the East China Sea and Yellow Sea has impacted the ecological niche. Global warming and escaped artificial breeding individuals have accelerated the expansion of the Tb population to this region. To protect the pure genetic lines of Tf and Tb, more stringent culture and management practices need to be implemented, and a larger-scale fishery resource survey will help to further explore introgression and hybridization between Tb and Tf.

### Diversified Reproductive Behaviors in *Takifugu*

Reproductive behavior includes any activity directed toward the perpetuation of a species, and the diversification of reproductive behavior will contribute to reproductive isolation and speciation (Chippindale, 2013). In species that have arisen from adaptive radiation due to the quick selection in different ecological environments, postzygotic isolation is not prevalent (Salzburger et al., 2002). Hence, prezygotic isolation caused by geographic isolation and different reproductive behaviors plays an important role in speciation during adaptive radiation. About 20 *Takifugu* species are widely distributed in the coastal of China, with only a few species exhibiting geographical isolation (Zhang and He, 2008). However, diversified reproductive behaviors, including mating period, spawning ground, and hatching conditions, accelerate speciation in *Takifugu*.

In this study, we identified several candidate genes, related to the semilunar reproduction rhythm, which had undergone selective sweep in Tn. As a widely distributed species, the geographical conditions promote natural hybridization of Tn with many *Takifugu* species, however, special reproductive behaviors of Tn lead to reproductive isolation. In addition to the specific occurrence of the mating period during the rising tides, Tn spawns only in the intertidal zone, which further reduces the possibility of hybridization with other species. Although we do not know how these specialized reproductive behaviors have developed, there is no doubt that they accelerates speciation in *Takifugu*. This rapid pattern of speciation also exists in *Platichthys flesus* (Momigliano et al., 2017) and stickleback (Berner et al., 2017). For example, *Platichthys flesus* in the Baltic Sea exhibit two reproductive behaviors: pelagic and demersal spawning. Since the low salinity of the Northern Baltic Sea cannot support the buoyancy of the egg, only demersal spawning flounders can thrive in this region. Molecular evidence showed that demersal and pelagic flounders were two ecological species that had arisen from a recent event of speciation, approximately 2,400 generations ago, and this speciation was faster than most known cases of ecological speciation in marine vertebrates (Momigliano et al., 2017). Migration during the mating period is another important reproductive behavior exhibited by *Takifugu* species. Toc migration has also been reported in the estuary along the Chinese coast from East China Sea to the Yangzi River for spawning during spring (Yang and Chen, 2008). Although other *Takifugu* species also migrate to the coastal area during the spawning season, they do not spawn in the freshwater river and rarely mate with To and Toc (Yasuji et al., 1991). On the other hand, a previous study showed that ecological shifts caused by colonization of and migration to estuarine habitats promote rapid adaptive divergence and reproductive isolation in incipient ecological species (Beheregaray and Sunnucks, 2001). Therefore, we speculate that the spawning behaviors of *Takifugu* species may be one of the driving forces of adaptive radiation, which is caused by changes in the estuarine environment during the glacial period. Hence, in future studies, we plan to reconstruct the demographic changes and historical population size of different *Takifugu* species, and then infer the geological events that lead to their differentiation, along with the period of occurrence, on a larger scale. In addition, we also plan to observe the reproductive behavior of more *Takifugu* species in the wild, which will provide more information for the study of speciation in *Takifugu*.

## Conclusion

This study focused on the phylogenetic relationships, introgression, hybridization, adaptive evolution, and demographic history of *Takifugu* species in East Asia. We, except for the Tp-Ta sister group, reliably confirmed the sister-groups of Tb-Tf, Tob-Tn, Tr-To, Tx-Toc, based on 4DTv sites. Combined with the phylogenetic tree constructed on the basis of the mitochondrial genome sequence, we identified four hybridized individuals who are a product of a mating between male Tb and female Tf. Further, Tn may be a potential hybrid of To and the Tx-Toc sister group. Admixture analysis of all individuals showed a divergence of the To population from other species and a bidirectional gene flow between Tb and Tf. Using the species-specific highly differentiated sites of homozygous genetic variation between the “purebred” of Tb and Tf, we detected low levels of introgression in seven “purebred” Tf individuals, and no introgressive signal was detected in “purebred” Tb individuals, indicating that an asymmetric introgression existed at the East China Sea and southern Taiwan Strait. This phenomenon may be caused by the huge pressure exerted by fisheries, global warming, and individuals escaping from artificial breeding enclosures. Homozygous sites of complete differentiation (*F*_st_ = 1) between To and the Tx-Toc sister group revealed numerous differences in their genetic origin, which is a result of autosomal recombination after potential introgression. As a set of typical species that have arisen from adaptive radiation, *Takifugu* also exhibits diverse phenotypes and behaviors. On a genome-wide scale, we detected two copies of *syt1*, *zar1* and *tgfbr1*, that are related to the semilunar reproduction rhythm in Tn and are involved in memory formation, embryo maturation, and the female reproductive system. Furthermore, we found that many genes regulating the reproductive rhythm and endocrine hormone levels in Tn had specific mutations in the CDS. Our research provides a reliable genetic resource of the phylogenetic relationships, evolutionary history, adaptive radiation, and hybridization in different *Takifugu* species. Additionally, our results can be used as a reference for the formulation of protection and release policies in artificial fisheries.

## Data Availability Statement

The datasets presented in this study can be found in online repositories. The names of the repository/repositories and accession number(s) can be found in the article/Supplementary Material.

## Ethics Statement

The animal study was reviewed and approved by Fujian Key Laboratory of Genetics and Breeding of Marine Organisms, College of Ocean and Earth Sciences, Xiamen University.

## Author Contributions

PX and BL conceived the project. BL contributed to the funding acquisition. ZZ wrote the manuscript. ZZ, YB, and JY performed the analysis and designed the charts and tables. BL conducted the sampling collection. ZZ, FP, and YS conducted the Re-sequencing libraries. All authors have validated and approved the manuscript.

## Conflict of Interest

The authors declare that the research was conducted in the absence of any commercial or financial relationships that could be construed as a potential conflict of interest.

## Footnotes

1 https://blast.ncbi.nlm.nih.gov/Blast.cgi

## Supplementary Material

Supplementary Figure 1 The photo and distribution of *T. poechilonotus*, *T. alboplumbeus*. **(A)** The photo of *T. poechilonotus*. **(B)** The photo of *T. alboplumbeus*. **(C)** The distribution of *T. poechilonotus*. **(D)** The distribution of *T. alboplumbeus*.

Supplementary Figure 2 The coefficient of variation of *K* in admixture from 3 to 18. The dotted line represents the best *K* value. CV, coefficient of variation; K, the number of groups.

Supplementary Figure 3 The distribution of *T. bimaculatus* (Tb) and *T. flavidus* (Tf) in the coastal of China. Brown represents the habitat of Tb, orange represents the habitat of Tf, red represents the coexist region of Tb and Tf, gray represents the unknown region.

Supplementary Figure 4 **(A)** The species-specific SNPs genotype of four hybrid individuals, Tb-specific and Tf-specific on mitochondrial genome. **(B)** The species-specific SNPs genotype of four hybrid individuals, Tb-specific and Tf-specific on autosomes.

Supplementary Figure 5 **(A)** Admixture analysis based on 129 genetic variation sites on the mitochondrial genome between Tb and Tf. **(B)** Admixture analysis based on 135 genetic variation sites on the mitochondrial genome between Tb and Tf. The orange represented the genetic group of Tf, The claret represented the genetic group of Tb.

Supplementary Figure 6 The hybrid diagram of Tb and Tf.

Supplementary Figure 7 The distribution of homozygous and complete differentiation genetic variance sites between Tob and Tx-Toc group in seven *Takifugu* species. **(A)** The distribution of homozygous genetic variance sites in seven *Takifugu* species. **(B)** The distribution of heterozygous genetic variance sites in seven *Takifugu* species. **(C)** The distribution of genetic variance sites on the mitochondrial genome in seven *Takifugu* species.

Supplementary Figure 8 The fine mapping of complete differentiation genetic variance sites between Tob and Tx-Toc group based on allele frequency of Tn. The dark blue plot represents the Tob-specific allele; the light blue plot represents the Tx-Toc specific allele.

Supplementary Figure 9 The fine mapping of complete differentiation genetic variance sites between Tx and Toc group based on allele frequency of Tn. The dark blue plot represents the Tx-specific allele; the light blue plot represents the Toc specific allele.

Supplementary Table 1 The statistics of sample size, collection location and accession number of sequencing data that download from NCBI database.

Supplementary Table 2 The statistics of sequencing data of 70 wild *Takifugu* individuals.

Supplementary Table 3 The statistics of sequencing data of 52 *Takifugu* individuals download from the NCBI database.

Supplementary Table 4 Statistics of genotyping of 122 *Takifugu* species.

Supplementary Table 5 Statistics of genotyping of 10 *Takifugu* species.

Supplementary Table 6 Statistics of genetic variance sites.

Supplementary Table 7 Statistics of the number of homozygous and complete differentiation genetic variance sites between Tob and Tx-Toc group in seven *Takifugu* species.

Supplementary Table 8 The significant selected regions of Tn based on the *F*_st_ threshold (*F*_st_ > 0.225).

Supplementary Table 9 The significant selected regions of Tn based on the π Ratio threshold (| Log_2_($\bar{\pi \,x/\pi \,T\,n}$)| > 1.5).

Supplementary Table 10 Mutations that in the exon of four candidate genes of Tn.

Supplementary Table 11 Mutations that in the exon of rhythm and sex hormones related genes of Tn.
