# Supplementary material for: Genome-Scale Phylogenetic and Population Genetic Studies Provide Insight Into Introgression and Adaptive Evolution of Takifugu Species in East Asia
Source: Front Genet. 2021 Feb 22;12:625600. doi: 10.3389/fgene.2021.625600 (PMC7937929; doi:10.3389/fgene.2021.625600)
Supplement: Supplementary Figure 1 — The photo and distribution of T. poechilonotus, T. alboplumbeus. (A) The photo of T. poechilonotus. (B) The photo of T. alboplumbeus. (C) The distribution of T. poechilonotus. (D) The distribution of T. alboplumbeus. [file Data_Sheet_1.PDF]

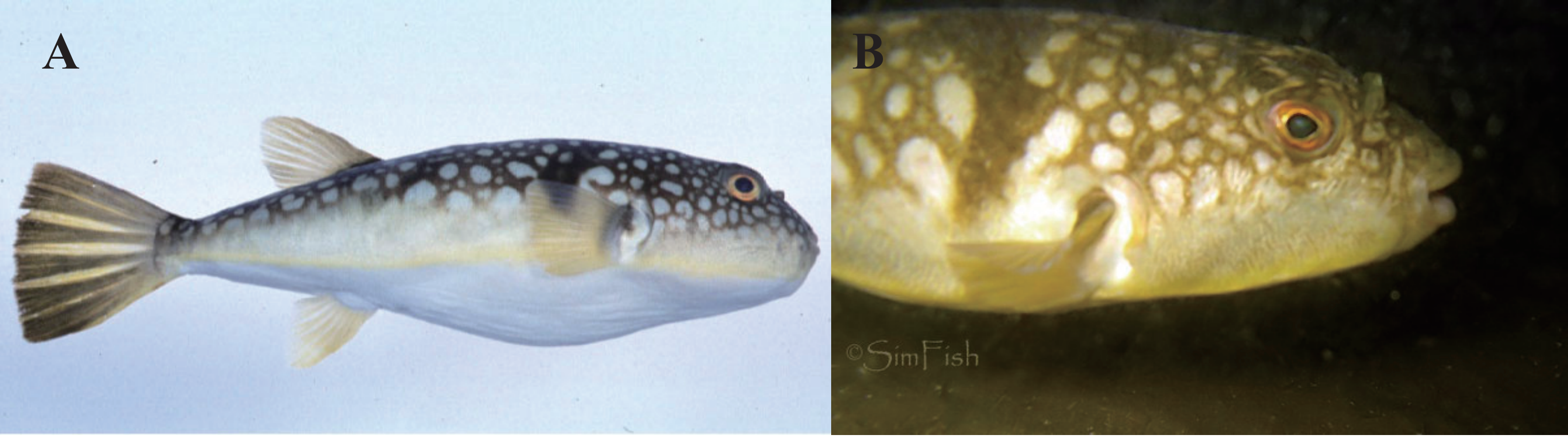

Takifugu poechilonotus ***Takifugu alboplumbeus***

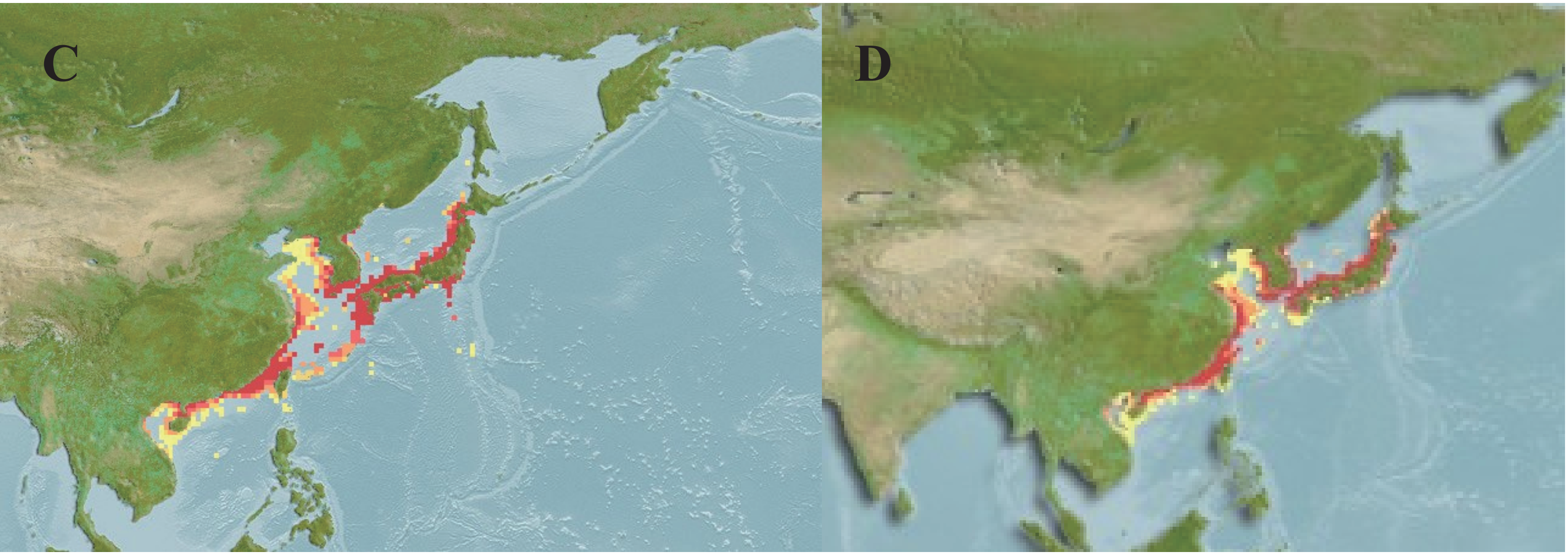

The *Takifugu* pictures were downloaded from fishbase (https://www.fishbase.us/)
The native range maps of Tp and Ta were downloaded from aquamaps (https://www.aquamaps.org/)
