# Supplementary figures and images for: Genome-Scale Phylogenetic and Population Genetic Studies Provide Insight Into Introgression and Adaptive Evolution of Takifugu Species in East Asia
Source: Front Genet. 2021 Feb 22;12:625600. doi: 10.3389/fgene.2021.625600 (PMC7937929; doi:10.3389/fgene.2021.625600)

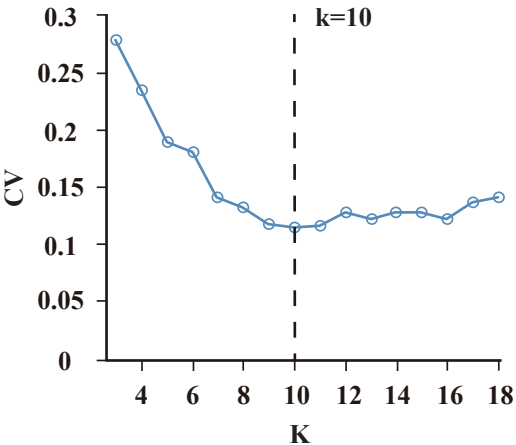

**CV: Coefficient of Variation**

**K: The Number of groups**

Supplement: Supplementary Figure 2 — The coefficient of variation of K in admixture from 3 to 18. The dotted line represents the best K value. CV, coefficient of variation; K, the number of groups. [file Data_Sheet_2.PDF]

40° N

35° N

30° N

25° N

- 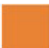 **Tf Habitat**
- 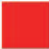 **Coexist**
- 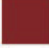 **Tb Habitat**
- 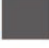 **Unknown**

110°E

115°E

120° E

125° E

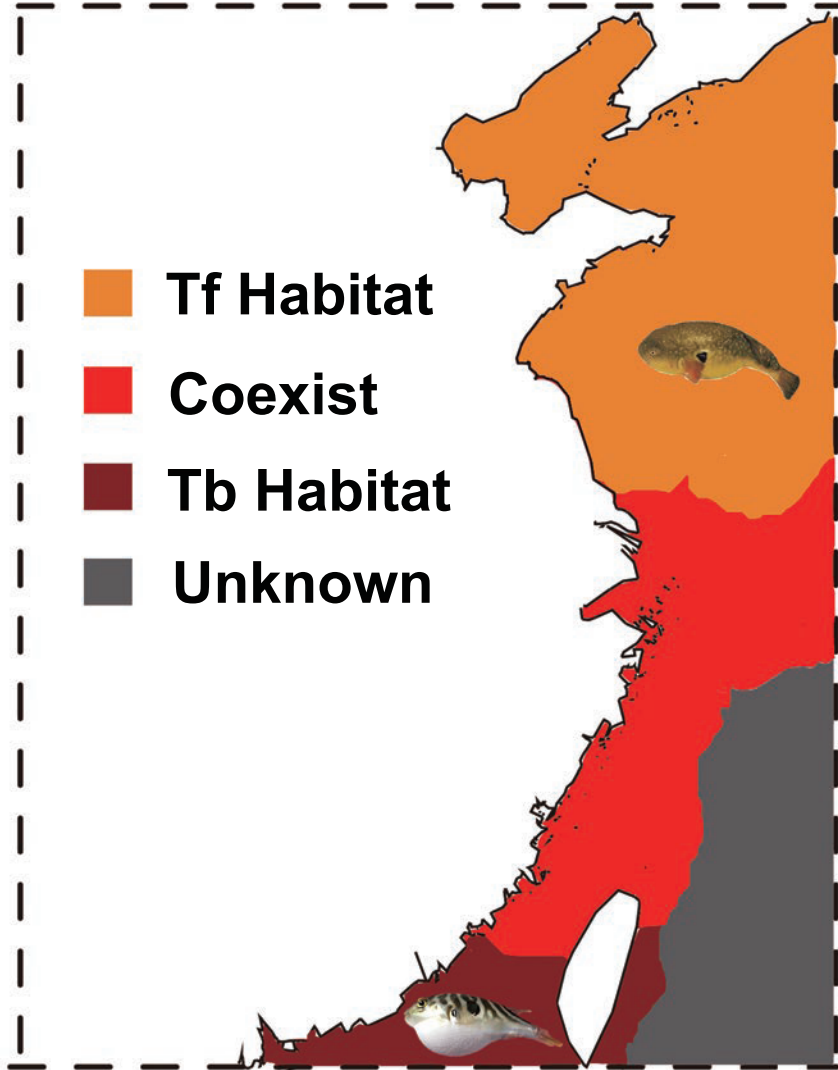

Supplement: Supplementary Figure 3 — The distribution of T. bimaculatus (Tb) and T. flavidus (Tf) in the coastal of China. Brown represents the habitat of Tb, orange represents the habitat of Tf, red represents the coexist region of Tb and Tf, gray represents the unknown region. [file Data_Sheet_3.PDF]

■ *Takifugu bimaculatus*  
■ *Takifugu flavidus*

A

Ancestry

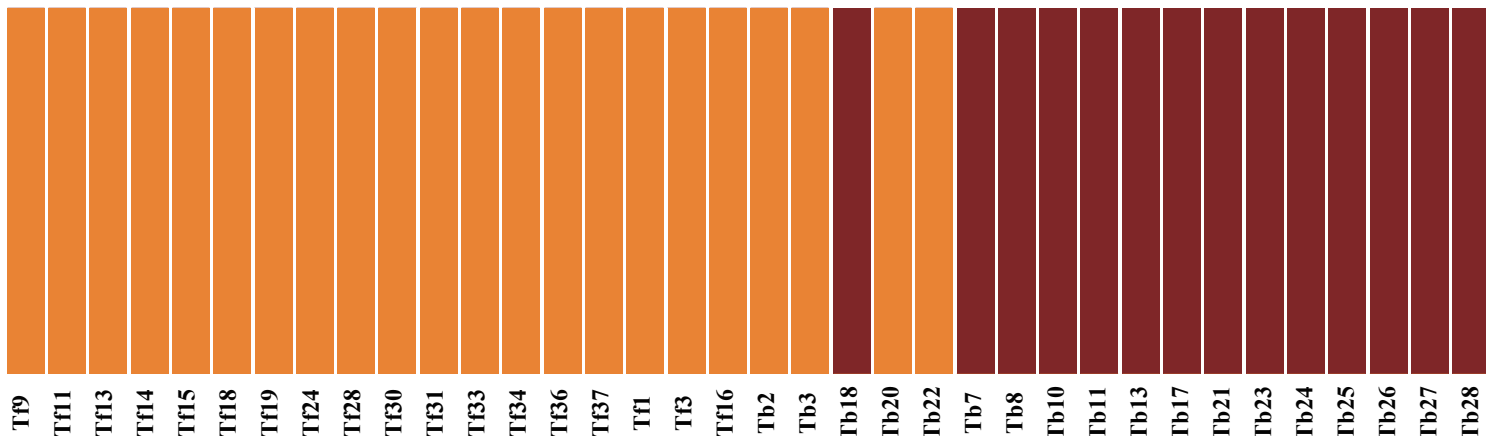

B

Ancestry

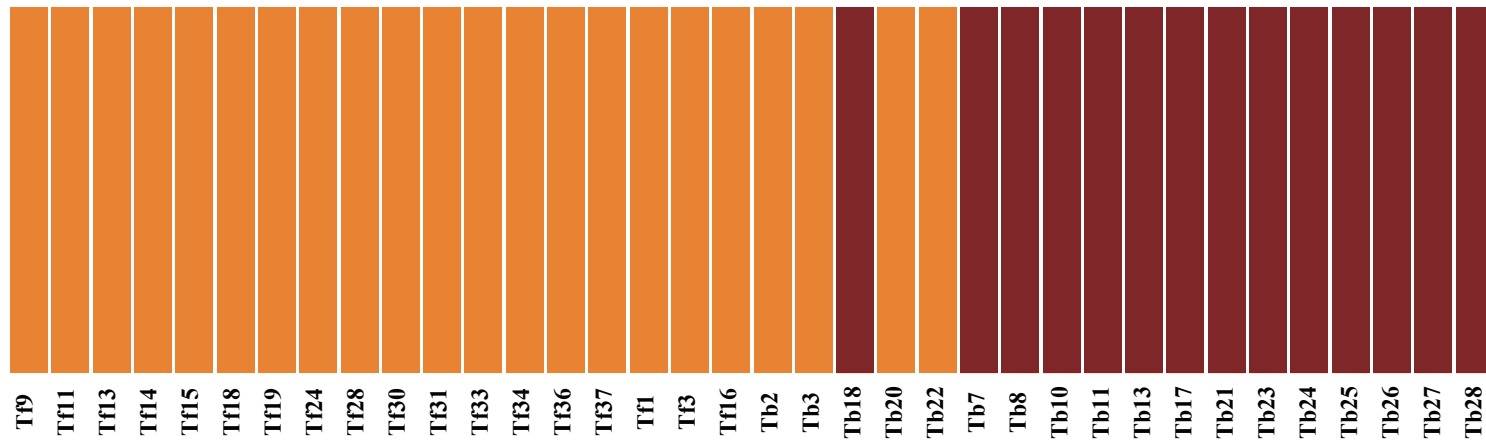

Individuals

Supplement: Supplementary Figure 5 — (A) Admixture analysis based on 129 genetic variation sites on the mitochondrial genome between Tb and Tf. (B) Admixture analysis based on 135 genetic variation sites on the mitochondrial genome between Tb and Tf. The orange represented the genetic group of Tf, The claret represented the genetic group of Tb. [file Data_Sheet_5.PDF]

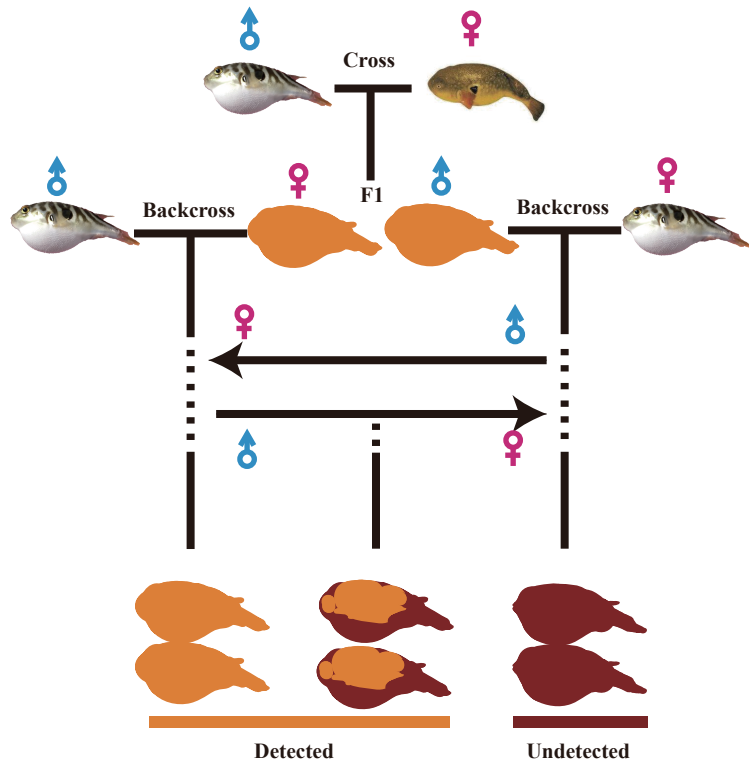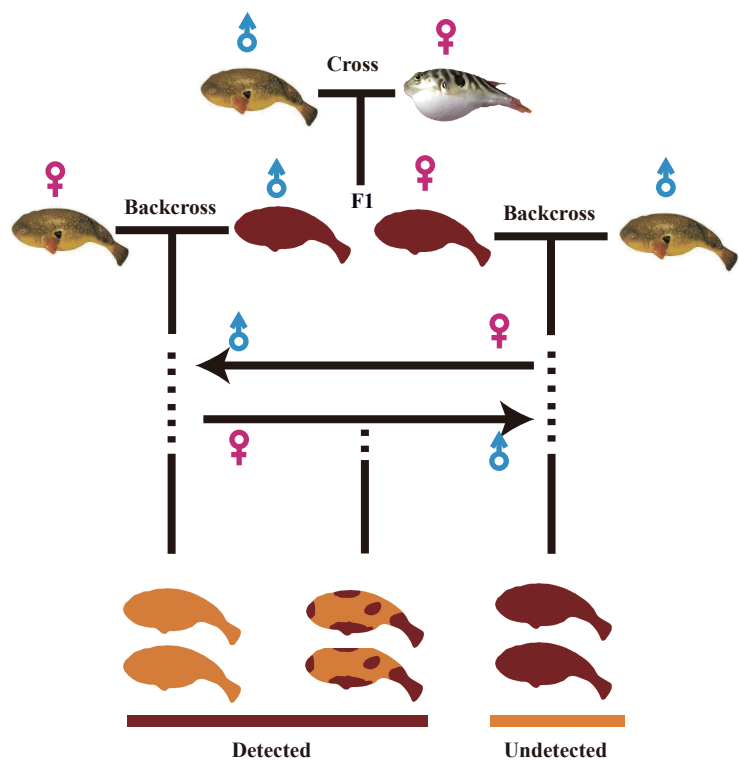

Supplement: Supplementary Figure 6 — The hybrid diagram of Tb and Tf. [file Data_Sheet_6.PDF]

A

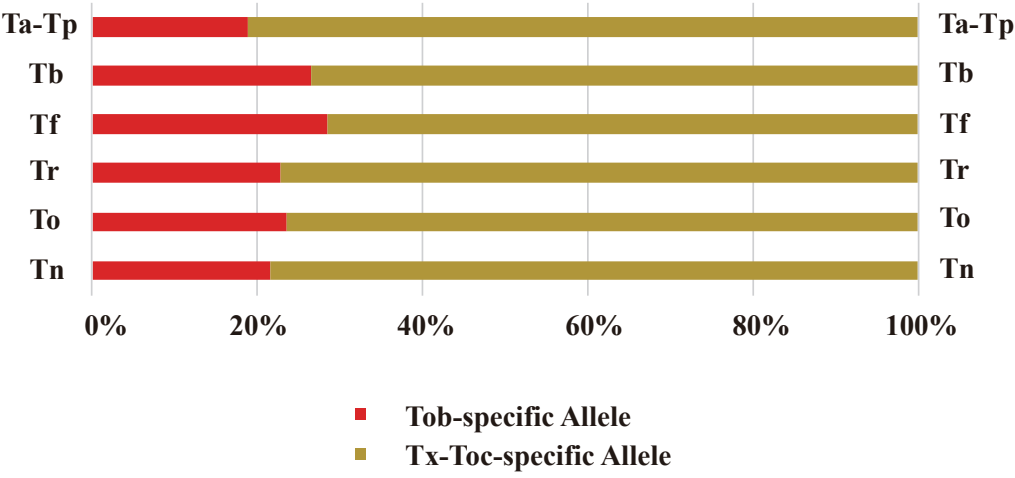

B

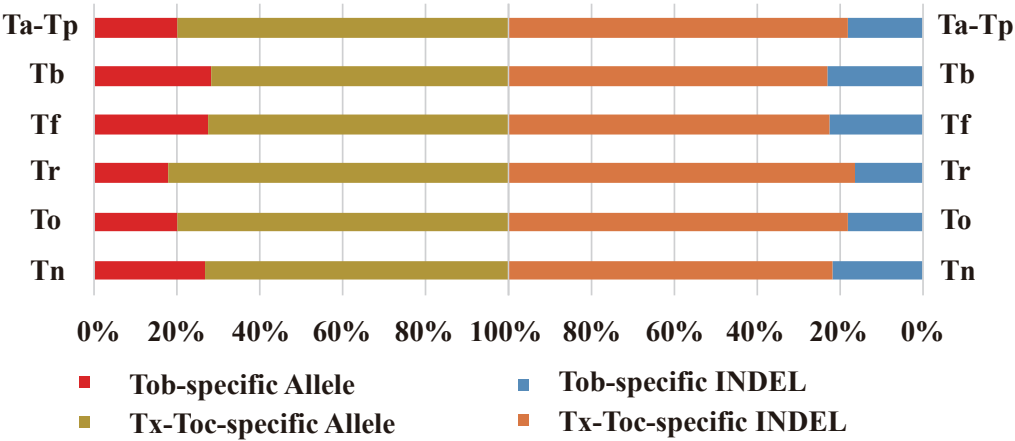

C

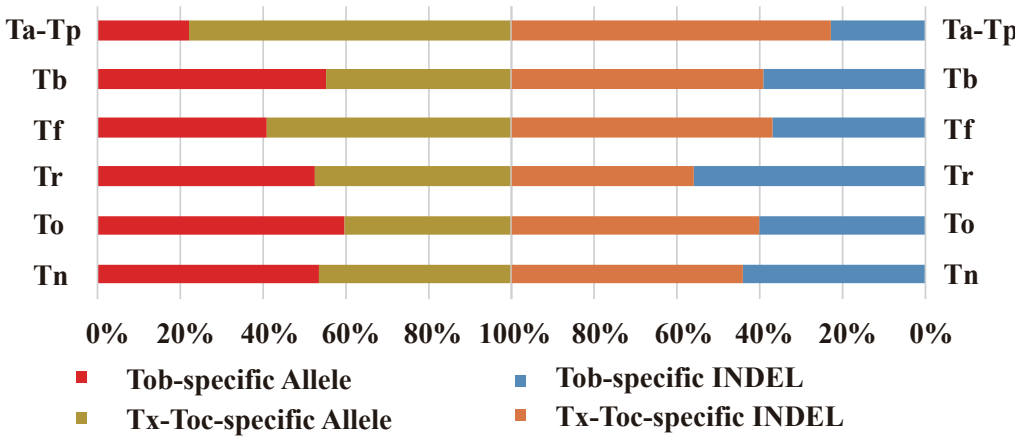

Supplement: Supplementary Figure 7 — The distribution of homozygous and complete differentiation genetic variance sites between Tob and Tx-Toc group in seven Takifugu species. (A) The distribution of homozygous genetic variance sites in seven Takifugu species. (B) The distribution of heterozygous genetic variance sites in seven Takifugu species. (C) The distribution of genetic variance sites on the mitochondrial genome in seven Takifugu species. [file Data_Sheet_7.PDF]

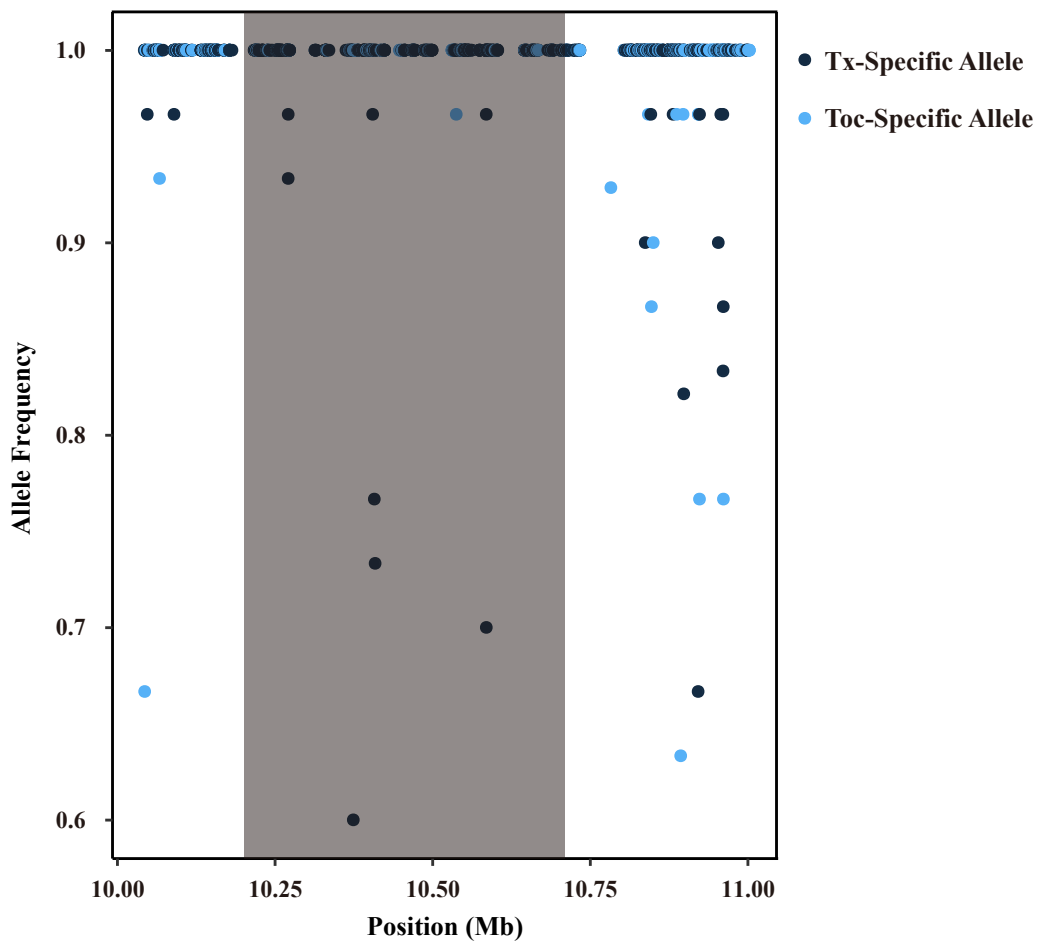

Supplement: Supplementary Figure 9 — The fine mapping of complete differentiation genetic variance sites between Tx and Toc group based on allele frequency of Tn. The dark blue plot represents the Tx-specific allele; the light blue plot represents the Toc specific allele. [file Data_Sheet_9.PDF]
