# Supplementary material for: Genome-Scale Phylogenetic and Population Genetic Studies Provide Insight Into Introgression and Adaptive Evolution of Takifugu Species in East Asia
Source: Front Genet. 2021 Feb 22;12:625600. doi: 10.3389/fgene.2021.625600 (PMC7937929; doi:10.3389/fgene.2021.625600)
Supplement: Supplementary Figure 4 — (A) The species-specific SNPs genotype of four hybrid individuals, Tb-specific and Tf-specific on mitochondrial genome. (B) The species-specific SNPs genotype of four hybrid individuals, Tb-specific and Tf-specific on autosomes. [file Data_Sheet_4.PDF]

A

Tf	T	C	T	T	T	C	C	A	C	G	G	T	T	G	C	G	C	C	C	T	T	A	C	C	C
Tb_2	T	C	T	T	T	C	C	A	C	G	G	T	T	G	C	G	C	C	C	T	T	A	C	C	C
Tb_20	T	C	T	T	T	C	C	A	C	G	G	T	T	G	C	G	C	C	C	T	T	A	C	C	C
Tb_22	T	C	T	T	T	C	C	A	C	G	G	T	T	G	C	G	C	C	C	T	T	A	C	C	C
Tb_3	T	C	T	T	T	C	C	A	C	G	G	T	T	G	C	G	C	C	C	T	T	A	C	C	C
Tb	C	T	C	C	C	T	T	G	T	A	A	C	C	A	T	A	T	T	T	C	C	G	T	T	T

Tf	G	T	G	A	C	T	T	G	C	A	T	C	C	A	T	T	G	T	C	T	C	G	T	T	C
Tb_2	G	T	G	A	C	T	T	G	C	A	T	C	C	A	T	T	G	T	C	T	C	G	T	T	C
Tb_20	G	T	G	A	C	T	T	G	C	A	T	C	C	A	T	T	G	T	C	T	C	G	T	T	C
Tb_22	G	T	G	A	C	T	T	G	C	A	T	C	C	A	T	T	G	T	C	T	C	G	T	T	C
Tb_3	G	T	G	A	C	T	T	G	C	A	T	C	C	A	T	T	G	T	C	T	C	G	T	T	C
Tb	A	C	A	G	T	C	C	A	T	G	C	T	T	G	C	C	A	C	T	C	T	A	C	C	T

Tf	G	C	C	A	A	T	C	C	C	T	G	A	A	A	T	T	C	G	T	T	C	C	C	C	C
Tb_2	G	C	C	A	A	T	C	C	C	T	A	A	A	A	T	T	C	G	T	T	C	C	C	C	C
Tb_20	G	C	C	A	A	T	C	C	C	T	G	A	A	A	T	T	C	G	T	T	C	C	C	C	C
Tb_22	G	C	C	A	A	T	C	C	C	T	G	A	A	A	T	T	C	G	T	T	C	C	C	C	C
Tb_3	G	C	C	A	A	T	C	C	C	T	A	A	A	A	T	T	C	G	T	T	C	C	C	C	C
Tb	A	T	T	G	T	C	T	T	A	C	A	G	G	G	A	C	T	A	C	C	T	T	T	T	T

Tf	T	C	G	C	C	G	C	T	T	A	A	G	T	A	T	G	C	A	C	C	A	A	T	T	A
Tb_2	T	C	G	C	C	G	C	T	T	A	A	G	T	A	T	G	C	A	C	C	A	A	T	T	A
Tb_20	T	C	G	C	C	G	C	T	T	A	A	G	T	A	T	G	C	A	C	C	A	A	T	T	A
Tb_22	T	C	G	C	C	G	C	T	T	A	A	G	T	A	T	G	C	A	C	C	A	A	T	T	A
Tb_3	T	C	G	C	C	G	C	T	T	A	A	G	T	A	T	G	C	A	C	C	A	A	T	T	A
Tb	C	T	A	A	T	A	T	C	C	G	G	A	C	G	C	A	T	G	T	T	G	G	C	C	G

Tf	T	C	C	T	T	T	T	T	C	C	T	G	T	T	C	T	C	G	T	A	A	T	C	T	G
Tb_2	T	C	C	T	T	T	T	T	C	C	T	G	T	T	C	T	C	G	T	A	A	T	C	T	G
Tb_20	T	C	C	T	T	T	T	T	C	C	T	G	T	T	C	T	C	G	T	A	A	T	C	T	G
Tb_22	T	C	C	T	T	T	T	T	C	C	T	A	T	T	C	T	C	G	T	A	A	T	C	T	G
Tb_3	T	C	C	T	T	T	T	T	C	C	T	G	T	T	C	T	C	G	T	A	A	T	C	T	G
Tb	C	T	T	C	C	C	C	C	T	T	C	A	C	C	T	C	T	A	C	T	G	C	T	C	A

Tf	A	G	T	G	A	C	C	A	T	C	A	G	A	A	G	C	C	G							
Tb_2	A	G	T	G	A	C	C	A	T	C	A	G	A	A	G	C	C	G							
Tb_20	A	G	T	G	A	C	C	A	T	C	A	G	A	A	G	C	C	G							
Tb_22	A	G	T	G	A	C	C	A	T	C	A	G	A	A	G	C	C	G							
Tb_3	A	G	T	G	A	C	C	A	T	C	A	G	A	A	G	C	C	G							
Tb	G	A	C	A	G	T	T	G	C	T	G	A	G	G	A	T	T	A							

B

Tf	T/T	A/A	C/C	A/A	T/T	C/C	A/A	G/G	A/A	C/C	C/C	C/C	C/C	G/G	A/A
Tb_2	T/T	G/G	G/G	A/G	A/A	T/T	G/G	G/A	A/G	T/T	T/T	T/T	T/T	A/A	T/T
Tb_20	C/C	G/G	C/G	G/G	T/A	C/T	A/G	A/A	G/G	C/T	C/T	C/T	C/C	A/A	T/T
Tb_22	C/C	G/G	G/G	G/G	A/A	T/T	G/G	A/A	G/G	T/T	T/T	T/T	T/T	G/A	A/T
Tb_3	C/C	A/G	G/G	G/G	A/A	T/T	G/G	A/A	G/G	C/T	C/T	C/T	T/T	A/A	T/T
Tb	C/C	G/G	G/G	G/G	A/A	T/T	G/G	A/A	G/G	T/T	T/T	T/T	T/T	A/A	T/T
