# Supplementary material for: Genome-Scale Phylogenetic and Population Genetic Studies Provide Insight Into Introgression and Adaptive Evolution of Takifugu Species in East Asia
Source: Front Genet. 2021 Feb 22;12:625600. doi: 10.3389/fgene.2021.625600 (PMC7937929; doi:10.3389/fgene.2021.625600)
Supplement: Supplementary Figure 8 — The fine mapping of complete differentiation genetic variance sites between Tob and Tx-Toc group based on allele frequency of Tn. The dark blue plot represents the Tob-specific allele; the light blue plot represents the Tx-Toc specific allele. [file Data_Sheet_8.PDF]

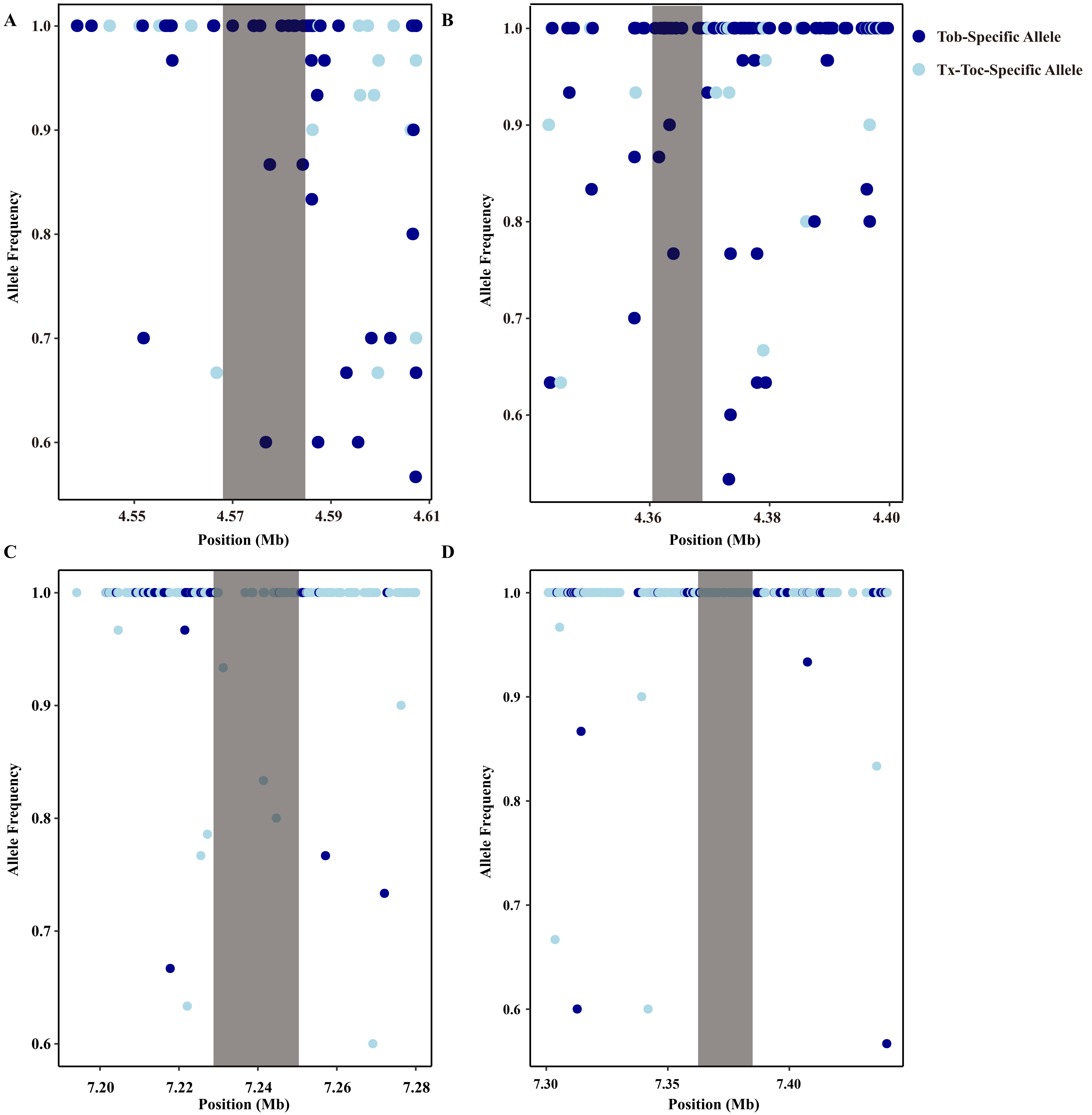

A
B
C
D
Tob-Specific Allele
Tx-Toc-Specific Allele
Allele Frequency
Position (Mb)
1.0
0.9
0.8
0.7
0.6
4.55
4.57
4.59
4.61
4.36
4.38
4.40
7.20
7.22
7.24
7.26
7.28
7.30
7.35
7.40
